# Trajectory changes of clinical empathy ability of Chinese nursing interns: A longitudinal study using latent transition analysis

**DOI:** 10.1097/MD.0000000000049054

**Published:** 2026-05-29

**Authors:** Mengmeng Hu, Xin Liu, Li Zhou, Hongjuan Chang, Chun Zhang

**Affiliations:** a China Resources & WISCO General Hospital, Wuhan, Hubei, China; b Wuhan University of Science and Technology Medical School, Wuhan, Hubei, China; c The Third People’s Hospital of Hubei Province, Wuhan, Hubei, China.

**Keywords:** emotional intelligence, empathy, latent profile analysis, latent transition analysis, nursing interns, psychological growth

## Abstract

This study utilized a longitudinal design and latent transition analysis to examine the distinct profiles, transitional trajectories, and influencing factors of clinical empathy among nursing interns. An 8-month follow-up survey, initiated in June 2024, was conducted among 762 nursing interns from 2 Grade A tertiary teaching hospitals in Hubei Province, China. Results identified 4 discrete latent profiles: low empathy-stable, low empathy-increasing, high empathy-stress, and high empathy-distress. The low empathy-stable profile demonstrated the lowest stability (7% maintenance probability) and a significant tendency to transition to the high empathy-stress profile (43% probability). In contrast, the maintenance probabilities for the remaining profiles ranged from 18% to 44%. Multivariate logistic regression revealed that gender, physical exercise, peer relationships, and reading empathy-related literature significantly predicted transition pathways (*P* < .05). Additionally, emotional intelligence served as a significant positive moderator in empathy development (*P* < .05). These findings highlight the dynamic nature of empathic capacity during clinical practicums. To promote long-term career development and prevent emotional exhaustion, hospitals and nursing schools should implement tailored emotional intelligence interventions, particularly for interns within the stress and distress-dominant empathy profiles.

## 1. Introduction

Within the framework of the biopsychosocial model, health transcends the mere absence of disease; rather, it is conceptualized as a holistic state of optimal physical, psychological, and social well-being.^[1]^ Modern nursing practice has imposed deeper requirements on nursing professionals. In addition to providing basic care, nurses are expected to offer psychological support to patients.^[2]^ Empathy refers to the capacity to understand and resonate with the emotions, thoughts, and needs of others, thereby generating analogous emotional experiences. It is conceptualized as a psychological process wherein an individual assumes the perspective of another, comprehends their viewpoints, and vicariously experiences their emotions.^[3]^ Empathy is a cornerstone for fostering harmonious nurse–patient relationships and is one of the essential qualities that nursing personnel should possess.^[4]^ The empathic abilities of nurses can assist patients in better managing the psychological stress induced by illness, enhance their courage and confidence in confronting challenges, and serve as a vital psychological resource during the recovery process.^[5]^ For nursing interns, their empathic abilities significantly influence their future professional attitudes and the development of their role personalities.^[6]^

According to the emotional intelligence (EI) model proposed by Rivers and Brackett et al, EI is defined as an individual’s capacity to adaptively perceive, understand, and regulate the emotions of oneself and others, as well as to utilize emotions for effective problem-solving. High levels of EI enable individuals to mitigate psychological distress and buffer against the adverse effects of negative emotions.^[7]^ In the context of nursing education, EI is recognized as a vital psychological resource that underpins students’ clinical competence.^[8]^ Furthermore, a study by Aghajani et al^[9]^ involving nursing interns demonstrated that those with higher EI exhibit superior emotional awareness and management capabilities. This allows them to flexibly implement emotion regulation strategies and maintain appropriate emotional engagement. Conversely, nursing students with lower EI are more vulnerable to emotional exhaustion due to a deficiency in effective emotion regulation resources.

In accordance with the Regulations on Nurses in China, undergraduate nursing students are mandated to complete a minimum of 8 months of clinical practicum prior to undertaking the nursing professional qualification examination.^[10]^ This period extends beyond mere technical skills training; it constitutes a critical transitional phase from academic study to professional clinical practice. During this practicum, empathy serves as a fundamental prerequisite for cultivating therapeutic nurse–patient relationships and delivering personalized nursing interventions. Crucially, empathy is not a static disposition but rather a dynamic construct shaped by situational contexts. As nursing students systematically engage with clinical patients for the first time, their empathic capacity may exhibit nonlinear developmental trajectories. This variability is often driven by the complexities of real-world healthcare settings, repeated exposure to patient suffering, and discrepancies in available clinical support resources.

However, most prior research relies on cross-sectional analyses that treat empathy as a continuous construct, thereby obscuring unobserved population heterogeneity and distinct developmental pathways. Latent transition analysis (LTA) addresses this limitation by identifying categorical latent statuses of nursing students based on their empathic capacity, and by quantifying the transition probabilities between these statuses across the clinical practicum.^[11]^ This approach offers a nuanced perspective on the developmental dynamics of empathy. Accordingly, the present study utilizes LTA to delineate the latent profiles and transitional trajectories of empathy among Chinese nursing interns. By incorporating EI as a predictor, we further analyze its influence on these stage-sequential transitions. Ultimately, this research aims to yield robust, evidence-based insights to inform the design of targeted empathy cultivation strategies.

## 2. Method

### 2.1. Study design and participants

A convenience sample of 762 nursing interns was recruited from 2 tertiary hospitals in Wuhan, Hubei Province, as the study participants. The 2 testing periods were the first week of the clinical internship and the final week after the internship, respectively, with an 8-month longitudinal follow-up investigation conducted between them. The first testing period (T1) occurred in June 2024, yielding 766 valid participants. The second testing period (T2) took place in March 2025, resulting in 762 valid participants. Seven hundred sixty-two nursing students who completed both T1 and T2 were included in this study, achieving a follow-up rate of 99.4%. This study was reviewed and approved by the Ethics Committee of the Medical College of Wuhan University of Science and Technology (Approval No: 2023177). All participants gave their informed consent to participate in this study.

### 2.2. Research tools

#### 2.2.1. General information survey

Based on a comprehensive literature review, the researchers developed a questionnaire themselves, which encompasses basic information such as gender, age, peer relationships, menstrual cycle regularity, and whether books related to empathy have been read.

#### 2.2.2. Interpersonal Reactivity Index (IRI)

Empathy was assessed using the IRI, originally developed by Davis (1980),^[12]^ which has been extensively utilized to evaluate empathic capacity in medical and nursing populations. The present study employed a validated Chinese version of the scale, comprising 22 items distributed across 4 dimensions: perspective-taking, personal distress, fantasy, and empathic concern. Items are rated on a 5-point Likert scale. Higher aggregate scores indicate elevated levels of empathy among the participants.^[13]^ In the current study, the Cronbach α coefficients for the scale were 0.79 at baseline (T1) and 0.84 at follow-up (T2).

#### 2.2.3. Wong and Law Emotional Intelligence Scale

EI was assessed using the scale developed by Wong and Law.^[14]^ This instrument encompasses 4 dimensions: self-emotion appraisal, others’ emotion appraisal, use of emotion, and regulation of emotion, with each dimension comprising 4 items. Responses are rated on a 7-point Likert scale. Higher aggregate scores indicate elevated levels of EI among the participants. In the present study, the Cronbach α coefficients for this scale were 0.93 at baseline (T1) and 0.95 at follow-up (T2).

### 2.3. Testing procedure

After obtaining informed consent from all participants, the research team briefed the students on the study’s objectives and provided clear guidelines for survey completion. Data collection was conducted longitudinally across the respective clinical practicum groups. At baseline (T1), sociodemographic characteristics alongside measures of clinical empathy and EI were collected. At follow-up (T2), only the empathy and EI scales were readministered. During each data collection session, 2 nursing postgraduate research assistants provided standardized verbal instructions, ensuring that the interns completed the questionnaires independently. All surveys were retrieved on-site immediately upon completion to ensure data integrity.

### 2.4. Data measurement

Data management and preliminary descriptive analyses were performed using SPSS version 26.0. Subsequently, LTA was conducted via Mplus version 8.3 to identify the latent profiles and state transitions of the IRI. Model fit was evaluated using several established information criteria: the akaike information criterion, the Bayesian information criterion (BIC), and the sample-size-adjusted BIC, with lower values indicating superior model fit. Furthermore, entropy was used to assess classification precision; it ranges from 0 to 1, with values closer to 1 reflecting higher accuracy. Finally, the Lo–Mendell–Rubin adjusted likelihood ratio test and the bootstrapped likelihood ratio test were employed. Significant *P*-values (*P* < .05) for these tests signify that a *k*-class model provides a significantly better fit than a (*k*-1)-class model.^[15]^

## 3. Results

### 3.1. General demographic

The average age of the nursing interns included in the sample was 21.79 ± 2.042 years. There were 113 males (14.8%) and 649 females (85.2%). 301 were specialized degree holders (39.5%) and 461 had a bachelor degree (60.5%). 358 exercised regularly (47%), 404 did not exercise regularly (53%), 233 had regular menstrual cycles (35.9%), and 416 had irregular menstrual cycles (64.1%). 102 had poor relationships with classmates (13.4%), 660 had good relationships with classmates (86.6%), 120 read books about EI (15.7%), and 642 did not read books about EI (84.3%).

### 3.2. Common method deviation test

The Harman single-factor test was employed to assess the common method deviation. Unrotated principal component factor analysis was conducted on the measured item data collected at 2 time points. The results indicate that the variance explained by the first extracted common factor in both analyses is below the threshold of 40% (T1: 20.12%; T2: 30.4%). This suggests that this study has no significant common method bias issue.

### 3.3. Repeated measures analysis of variance

A repeated measures analysis of variance was conducted to examine the differences in the main variables at 2 time points.^[16]^ The results indicated that, in the IRI scale, significant differences were observed for opinion selection (*F* = 7.47, *P* = .001), personal distress (*F* = 3.671, *P* = .026), Fantasy (*F* = 18.429, *P* < .01), empathic concern (*F* = 24.647, *P* < .01), and the total score (*F* = 70.23, *P* < .01). In the EI Scale, significant differences were also found for self-emotion appraisal (*F* = 3.355, *P* = .047), regulation of emotion (*F* = 20.87, *P* < .01), use of emotion (*F* = 24.273, *P* < .01), others’ emotion appraisal (*F* = 23.75, *P* < .01), and the total score (*F* = 58.23, *P* < .01). See Table 1.

**Table 1:** Analysis of variance for repeated measurements at time points T1 and T2.

item	T1	T2	*F*	*P*
	*M* ± SD	*M* ± SD		
Perspective-taking	17.26 ± 6.015	15.67 ± 4.242	7.47	.001
Personal distress	8.87 ± 4.435	12.33 ± 4.685	3.671	.026
Fantasy	11.95 ± 3.707	11.39 ± 3.687	18.429	<.01
Empathic concern	12.91 ± 3.721	15.78 ± 4.109	24.647	<.01
Self-emotion appraisal	19.93 ± 4.779	20.34 ± 4.341	3.355	.047
Regulation of emotion	27.08 ± 7.271	18.42 ± 4.422	20.87	<.01
Use of emotion	9.97 ± 2.750	18.84 ± 4.502	24.273	<.01
Others’ emotion appraisal	19.36 ± 2.750	19.44 ± 4.508	23.75	<.01
IRI	46.74 ± 10.346	58.53 ± 14.494	70.23	<.01
WLEIS	76.34 ± 16.454	77.05 ± 14.733	58.23	<.01

IRI = Interpersonal Reactivity Index, WLEIS = Wong and Law Emotional Intelligence Scale.

### 3.4. Latent transition analysis

Using the response situations of the 4 dimensions from the T1 and T2 IRI Scales for Chinese nursing interns as initial models, one category was incrementally added each time. The fitting indicators of the resulting models were comprehensively analyzed to determine the optimal model. Five-category models were constructed at 2 time points (T1 and T2), and the fitting indices of each model were systematically compared. At the T1 time point, Model 4 demonstrated the best goodness-of-fit among all models, with an Entropy value of 0.886 (higher than Models 2, 3, and 5, with Entropy values of 0.851, 0.857, and 0.877, respectively). Additionally, the *P* values for the Lo–Mendell–Rubin adjusted likelihood ratio test and bootstraped likelihood ratio test were <.01, indicating statistical significance. Furthermore, the classification accuracy of Model 4 was more consistent with the actual situation, with an average probability of 93.0% across profile. At the T2 time point, the akaike information criterion, BIC, and sample-size adjusted BIC decreased progressively as the number of profile increased. The Entropy values of Models 4 and 5 were closest to 1 (0.896 and 0.925, respectively). However, when transitioning to a 5-category model, the Lo–Mendell–Rubin test results were no longer significant (*P* > .05). These findings suggest that Model 4 provides a better fit than Model 5, with an average probability of 94.0% across profile. See Table 2.

**Table 2:** The LTA model at time points T1 and T2.

Model	AIC	BIC	aBIC	Entropy	LMR	BLRT	Category probability
1	17,665.984	17,703.072	17,677.668	–	–	–	–
2	16,446.955	16,507.222	16,465.941	0.851	<0.01	<0.01	0.654/0.346
3	15,838.807	15,922.254	15,865.097	0.857	0.008	<0.01	0.502/0.304/0.194
**4**	**15,458.163**	**15,564.790**	**15,491.755**	**0.886**	**<0.01**	**<0.01**	**0.188/0.088/0.271/0.453**
5	15,234.459	15,364.265	15,275.353	0.877	0.08	<0.01	0.135/0.322/0.306/0.071/0.165
1	17,724.892	17,761.980	17,736.576	–	–	–	–
2	16,644.598	16,704.866	16,663.585	0.854	<0.01	<0.01	0.571/0.429
3	16,169.203	16,252.650	16,195.493	0.863	0.019	<0.01	0.396/0.422/0.182
**4**	**15,738.014**	**15,844.641**	**15,771.606**	**0.896**	**0.012**	**<0.01**	**0.189/0.321/0.347/0.142**
5	15,464.605	15,594.412	15,505.500	0.925	0.766	<0.01	0.180/0.297/0.336/0.158/0.029

Bold indicates the importance of the fourth model.

AIC = akaike information criterion, aBIC = adjusted Bayesian information criterion, BIC = Bayesian information criterion, BLRT = bootstrapped likelihood ratio test, LMR = Lo–Mendell–Rubin.

**Figure 1.**
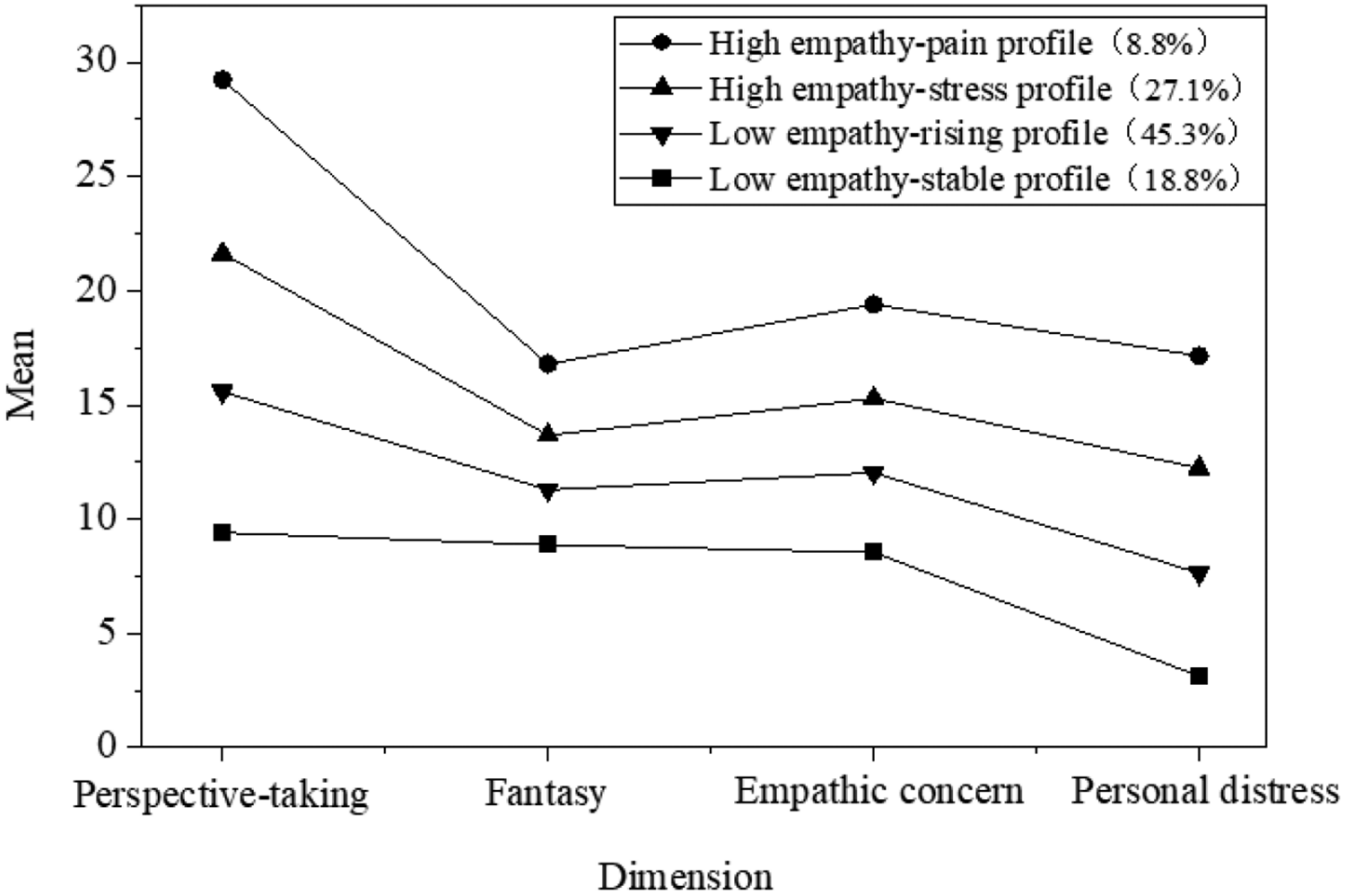
Categorization diagram of clinical empathy ability for Chinese nursing interns at Time Point T1.

**Figure 2.**
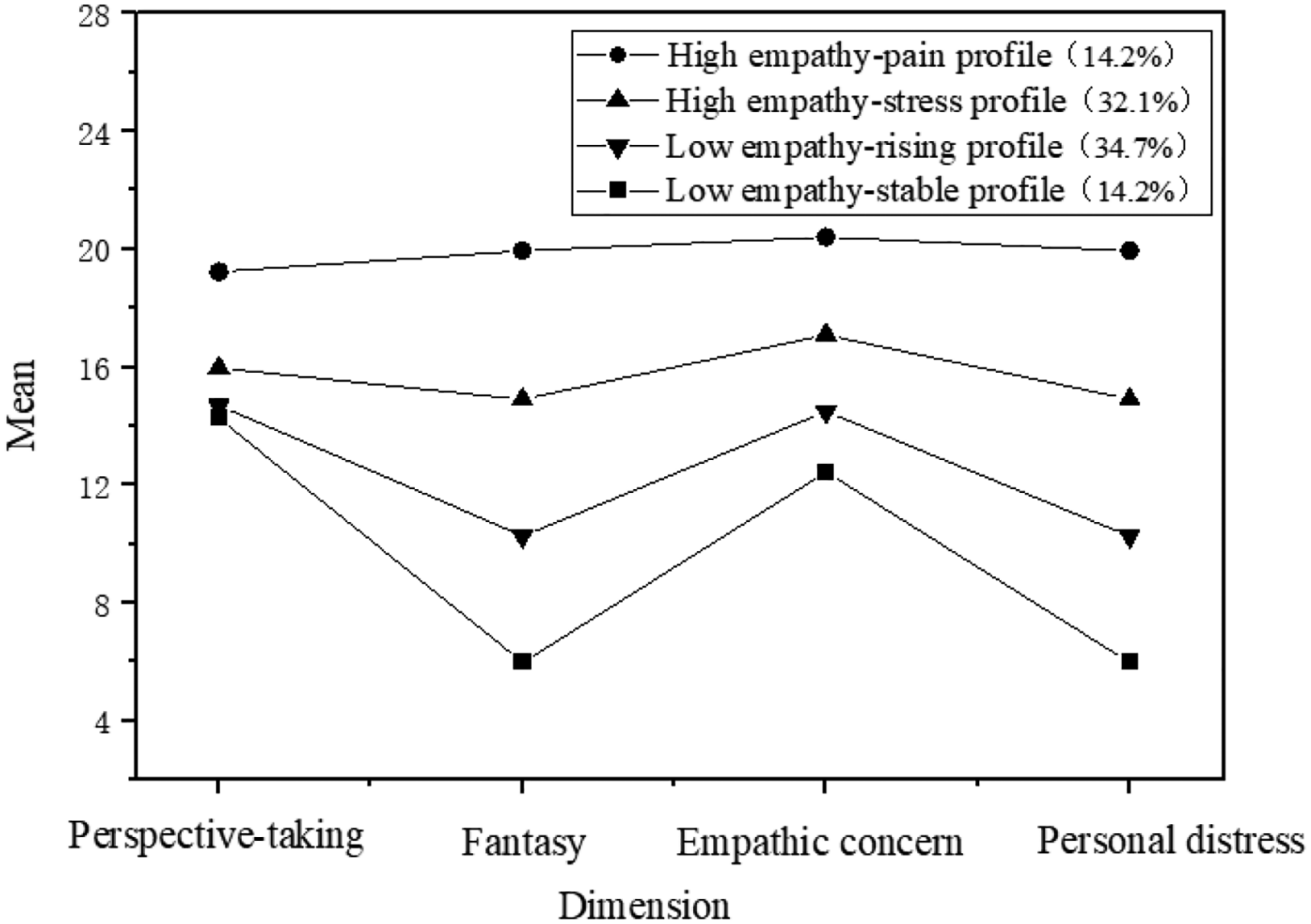
Categorization diagram of clinical empathy ability for Chinese nursing interns at Time Point T2.

According to the characteristics of the conditional mean distribution map across the 4 dimensions of the IRI scale, the 4 profile were systematically named. As illustrated in Figures 1 and 2, the mean values for each dimension of Profile 1 and 2 are relatively low and designated as “Low Empathy – Stable Profile” and “Low Empathy – Rising Profile,” respectively. For Profile 3 and 4, the mean values in opinion selection and personal distress gradually increase higher than those observed in other dimensions. Consequently, these profile are labeled “High Empathy – Stress Profile” and “High Empathy – Distress Profile,” respectively.

### 3.5. Latent transition analysis

LTA were performed on the 4 latent profile at time points T1 and T2, with the results presented in Table 3. The diagonal elements of the transformation matrix represent the probabilities that subjects remain in their original latent profile at different time points. Specifically, for the low empathy-stable category, the likelihood of remaining in this category is 7%. In contrast, the probabilities of transitioning to the low empathy-rising, high-empathy-stress, and high-empathy-distress profile are 14%, 43%, and 36%, respectively. The likelihood of maintaining this category for the low empathy-rising category is 18%, with transition probabilities to the low empathy-stable, high empathy-stress, and high-empathy-distress profile being 31%, 32%, and 19%, respectively. For the high-empathy-stress category, the likelihood of remaining in this category is 24%, with transition probabilities to the low empathy-stable, low empathy-rising, and high empathy-distress profile being 35%, 18%, and 24%, respectively. Finally, for the high-empathy-distress category, the probability of remaining in this category is 14%, with transition probabilities to the low empathy-stable, low-empathy-rising, and high empathy-stress profile being 27%, 19%, and 44%, respectively.

**Table 3:** Analysis of latent transition in clinical empathy ability among nursing students at time points T1 and T2.

Time	T2			
	Low empathy-stable	Low empathy-rising	High empathy-stress	High empathy -distress
T1				
Low empathy-stable	**0.07**	0.14	0.43	0.36
Low empathy-rising	0.31	**0.18**	0.32	0.19
High empathy-stress	0.35	0.18	**0.24**	0.24
High empathy-distress	0.27	0.15	0.14	**0.44**

Bold indicates the importance of the fourth model.

### 3.6. Influencing factors on the occurrence ratio and transformation probability

To investigate the effects of factors such as gender, exercise, regularity of menstrual cycles, peer relationships, reading empathetic literature, and EI on the latent profile of clinical empathy among nursing students during different internship stages, a multiple logistic regression analysis was conducted with the low empathy-stable profile serving as the reference category. The odds ratios (OR) were calculated to determine the ratios of probability changes for subjects transitioning into the low empathy-rising, high empathy-stress, and high empathy-distress profile relative to the low empathy-stable profile under the influence of covariates. The results are presented in Table 4. Male interns were less likely to belong to the low empathy-rising and high empathy-stress profile than female interns. Neither the frequency of exercise nor the regularity of menstrual cycles significantly affected the classification outcomes. Peer relationships, however, showed notable differences: compared to poor peer relationships, good peer relationships were associated with a higher probability of belonging to the high empathy-stress and high empathy-distress profiles. Additionally, interns who had not read empathetic literature were likelier to belong to the low empathy-rising and high empathy-stress profiles than those who had. Finally, interns with high EI were less likely to belong to the high empathy-stress and high empathy-distress profiles compared to those with low EI.

**Table 4:** The occurrence ratio of T1 potential probability under the influence of predictor variables.

Factors	Low empathy-rising type	High empathy-stress type	High empathy-pain type
Gender (0 = female, 1 = male)	0.699*	0.905*	1.236
Exercise (0 = No, 1 = Yes)	0.856	1.338	1.094
Regular menstrual cycle (0 = No, 1 = Yes)	0.769	1.259	1.972
Peer relationship (0 = poor, 1 = yes)	0.423	1.106*	1.807*
Empathetic literature reading (0 = No, 1 = Yes)	1.762**	2.113*	1.127
Emotional Intelligence	0.958	0.754**	0.213*

The regularity of menstruation is only investigated in females.

* *P* < .05.

** *P* < .01.

To investigate the influencing factors of latent category transformations in clinical empathy among nursing students during their internship period, variables such as gender, frequency of exercise, regularity of menstrual cycles, peer relationships, reading of empathy-related literature, and EI were incorporated as predictive variables in the latent class model. Subjects remaining in their original latent category were the reference group for multinomial logistic regression analysis. The results are presented in Table 4 below. An OR >1 indicates that the likelihood of a subject undergoing the specified transformation increases under the influence of the predictor variable; conversely, an OR <1 suggests a decrease in probability. According to Table 5, male nursing students exhibit a reduced likelihood of transitioning from the low empathy-stable profile to the low empathy-rising profile (OR = 0.685) and the high-empathy-stress profile (OR = 0.685) compared to female nursing students. Nursing students who exercise regularly demonstrate a decreased likelihood of transitioning from the low empathy-stable profile to the high empathy-stress profile (OR = 0.794) and the high empathy-distress profile (OR = 0.312) relative to those who do not exercise frequently. Students with positive peer relationships are more likely to transition from low empathy-ascending to high empathy-distressful (OR = 2.010) than those with poor peer relationships. Furthermore, nursing students who have read books on EI exhibit a higher probability of transitioning from the high empathy-stress profile to the low empathy-upward profile (OR = 1.025) than those who have not read such literature. EI also influences the likelihood of transitions: the probability of the low empathy-ascending profile transforming into the low empathy-stable profile increases (OR = 1.023), as does the possibility of the high empathy-stress profile transforming into the low empathy-stable profile (OR = 1.008).

**Table 5:** shows the occurrence ratio of the probability of changes in clinical empathy ability of intern nursing students under the influence of predictive variables.

Factors	Latent state	Low empathy-stable	Low empathy-rising	High empathy-stress	High empathy-distress
Gender (0 = female, 1 = male)	Low empathy-stable	REF	**0.684** *	**0.685** *	1.157
	Low empathy-rising	1.462	REF	1.264	1.686
	High empathy-stress	1.156	0.791	REF	1.334
	High empathy-distress	0.867	0.593	0.750	REF
Exercise (0 = No, 1 = Yes)	Low empathy-stable	REF	0.293	**0.794** *	**0.312** *
	Low empathy-rising	0.774	REF	1.387	1.015
	High empathy-stress	0.558	0.721	REF	0.732
	High empathy-distress	0.762	0.985	1.367	REF
Regular menstrual cycle (0 = No, 1 = Yes)	Low empathy-stable	REF	0.817	0.900	0.806
	Low empathy-rising	1.224	REF	1.101	0.987
	High empathy-stress	1.112	0.908	REF	0.896
	High empathy-distress	1.240	1.013	1.116	REF
peer relationship (0 = poor, 1 = yes)	Low empathy-stable	REF	1.001	1.158	2.012
	Low empathy-rising	0.999	REF	0.987	**2.010** *
	High empathy-stress	0.863	0.864	REF	1.736
	High empathy-distress	0.497	0.497	0.576	REF
Empathetic literature reading (0 = No, 1 = Yes)	Low empathy-stable	REF	0.960	0.976	1.109
	Low empathy-rising	1.041	REF	1.016	1.154
	High empathy-stress	**1.025** *	0.984	REF	1.136
	High empathy-distress	0.902	0.866	0.880	REF
WLEIS	Low empathy-stable	REF	1.016	0.991	1.007
	Low empathy-rising	**1.023** **	REF	1.017	1.009
	High empathy-stress	**1.008** *	0.983	REF	0.993
	High empathy-distress	1.016	0.991	1.007	REF

REF is the reference group. The reference category of the dependent variable is the subjects who retain the original group. Listed as a potential state of T1.

WLEIS = Wong and Law Emotional Intelligence Scale.

* *P* < .05.

** *P* < .01.

## 4. Discussion

This study utilized LTA to examine the latent states of clinical empathy among nursing students during their internship and the transformation patterns across time. Additionally, a systematic investigation was conducted into the effects of various factors, including gender, exercise habits, regularity of menstrual cycles, peer relationships, reading EI literature, and EI, on the transition model of empathy ability. The findings indicate that the clinical empathy of trainee nurses can be categorized into 4 distinct latent classes: low empathy-stable profile, low empathy-rising profile, high empathy-stress profile, and high empathy-distress profile. These results differ from the 3-class model of empathy proposed by Xie et al^[17]^ for undergraduate nursing students (high opinion selection-low personal distress group, low empathy ability group, and high empathy ability group). This discrepancy may arise from differences in research design and the unique stress characteristics inherent to clinical settings. Notably, LTA analysis uncovered the dynamic evolution of empathy during the clinical internship process. Under the high empathy dimension, influenced by internship-related stress, 2 subprofiles emerged: the high empathy-stress profile (characterized by short-term improvements in empathy accompanied by emotional burden) and the high empathy-distress profile (psychological exhaustion resulting from prolonged maintenance of high empathy levels). Importantly, nursing students in all 4 latent profile exhibited the highest scores in the opinion selection dimension across both measurement points, suggesting exceptional emotional perception abilities in clinical practice. As a critical component of empathy, opinion selection reflects an individual’s inclination to comprehend others’ psychological states actively.^[18]^ Clinical practice is a pivotal stage for nursing students transitioning into professional roles, where professional identity development drives them to deepen their understanding of patient needs. This role transformation may serve as a key driver for the manifestation of enhanced opinion selection ability.^[19]^

The analysis of latent transformation results indicates that the low empathetic-stable profile has the lowest probability of maintaining its original potential category (7%). In contrast, the high empathetic-distressful profile has the highest probability of maintaining its original potential category (44%). The low empathetic-stable profile is more likely to transform into the high empathetic-stress profile (43%) and the high empathetic-distressful profile (36%). These findings suggest clinical empathy among nursing students during their internship exhibits significant temporal dynamics. Unlike classroom simulations or theoretical learning, clinical practice demands that nursing students confront patients’ distress, anxiety, and even death directly over extended periods. This role transition and establishing professional identity contribute to fluctuations in nursing students’ empathy abilities.^[20]^ Dynamic systems theory also underscores that empathy is not a fixed trait but fluctuates based on environmental interactions.^[21]^ This study reveals an “upward trend” in the clinical empathy of trainee nurses during their internship period. High empathy significantly enhances patient satisfaction, improves treatment adherence, and reduces medical disputes.^[22,23]^ However, Marshman et al argue that excessive empathy among healthcare professionals can lead to self-depletion and emotional suffering,^[24]^ consistent with this study’s classification results. Individuals with excessive empathy are prone to somatic reactions such as anxiety, sleep disturbances, and physical distress.^[25]^ An animal experiment demonstrated^[26]^ that when monkeys observe similar distress in others, their mirror neurons activate distress-related brain regions (e.g., the insula and anterior cingulate cortex), simulating the emotional experience of others and causing involuntary frowning or tearing. Wang et al found that nursing students’ empathy abilities and job burnout are influenced by factors such as professional identity and clinical environmental stress. Long-term emotional involvement may diminish work enthusiasm and potentially lead to a desire to distance oneself from the medical field.^[27]^ Therefore, this study posits that empathy is a double-edged sword in the medical profession. Moderate empathy enhances the quality of care, while prolonged excessive empathy may result in psychological exhaustion, occupational risks, and physical symptoms. Schools and hospitals should guide nursing students to manage emotional boundaries scientifically, strengthen support systems, and ensure sustainable career development while preserving humanistic care.

Multivariate logistic regression analysis revealed that female nursing students who exercised infrequently exhibited high levels of peer support and had not read literature on empathy. This demonstrated a significant tendency toward subprofiles by high empathy traits. This finding aligns with most psychologists’ research, indicating that women score significantly higher than men in both emotional empathy (feeling others’ emotions) and cognitive empathy (understanding others’ perspectives).^[2,19]^ Gupta et al further highlighted that moderate exercise can regulate emotions and alleviate stress, indirectly influencing empathy. Conversely, female nursing students who do not exercise regularly may lack the emotional regulation and stress relief benefits of physical activity.^[28]^ Additionally, the study found that nursing students with high peer support were more susceptible to negative effects associated with heightened empathy abilities. highly empathetic nursing students might be more prone to perceiving and caring for patients’ negative emotions, leading to potential emotional exhaustion.^[29]^ To mitigate emotional depletion among highly empathetic-stress profile and highly empathetic-distress profile nursing students, interventions such as emotional teaching courses,^[30]^ mindfulness training,^[31]^ and self-care training^[32]^ could be encouraged.

EI is a protective factor in the low empathetic-stable and the low empathetic-rising profiles. Some highly empathetic Chinese nursing interns may experience excessive emotional empathy, insufficient cognitive empathy, and become overly immersed in others’ narratives. For such students, participation in emotional management training programs can be encouraged to develop their capacity for emotional regulation and enhance their EI. Ma Z et al^[33]^ demonstrated that simulating nurse–patient interaction scenarios through virtual reality and training selective empathy enables individuals to distinguish between their emotions and those of others, thereby avoiding over-involvement or ineffective assistance. Additionally, required nursing students to document “the emotions of others I perceived” and “my own needs understood” daily, fostering self-awareness through comparative reflection and preventing excessive self-sacrifice. A daily 10-minute mindfulness practice can also assist nursing students in efficiently disengaging from emotional residues following empathetic engagement.^[34]^ Furthermore, maintaining a healthy lifestyle, encompassing regular routines, a balanced diet, and moderate exercise, significantly contributes to nursing students’ emotional and intellectual development.^[35]^ Consequently, enhancing EI facilitates improved communication with patients and healthy interpersonal relationships, promotes a more precise self-understanding, strengthens self-awareness and self-regulation abilities, and enables nursing students to better adapt to future professional environments.

## 5. Conclusion and limitations

The primary innovation of this study lies in its comprehensive and dynamic exploration of the latent profile and transformation characteristics of clinical empathy among nursing interns. Specifically, it investigates how demographic factors and EI influence the transformation of these profile. Nevertheless, certain limitations and deficiencies in this research warrant further investigation in future studies. First, this study only examined a limited set of variables affecting clinical empathy, while many other significant influencing factors were not included. Second, although a longitudinal design was employed to explore the temporal development of clinical empathy subprofiles among nursing interns, future studies could benefit from incorporating samples with diverse professional and cultural backgrounds to examine the similarities and differences in the development and changes of empathy across various contexts.

## Acknowledgments

We thank the nursing students who participated the study and completed questionnaire forms.

## Author contributions

**Data curation:** Xin Liu, Li Zhou.

**Writing – original draft:** Mengmeng Hu.

**Writing – review & editing:** Hongjuan Chang, Chun Zhang.
